# Hydrocarbon Signatures of the Ectoparasitoid *Sphecophaga vesparum* Shows Wasp Host Dependency

**DOI:** 10.3390/insects11050268

**Published:** 2020-04-28

**Authors:** Cintia Akemi Oi, Robert L. Brown, Ian Stevens, Tom Wenseleers

**Affiliations:** 1 Laboratory of Socioecology and Social Evolution, KU Leuven, 3000 Leuven, Belgium; 2 Manaaki Whenua—Landcare Research, P.O. Box 69040, Lincoln 7640, New Zealand

**Keywords:** chemical mimicry, cuticular hydrocarbons, vespid wasps, *Sphecophaga vesparum*, Ichneumonidae

## Abstract

*Sphecophaga vesparum* often parasitizes nests of vespid wasps such as *Vespula vulgaris* and *Vespula germanica*. Inside the colonies, the ectoparasitic larvae feed on the immature forms of the wasps. There are two adult forms of *S. vesparum*. The large, winged adults emerge from either rigid yellow cocoons or the orange cocoons used for overwintering. The small, brachypterous females emerge from soft, white cocoons. The species is facultative deuterotokous, producing mostly parthenogenic females and infrequently producing males. Here, we describe the production of chemical compounds related to the different developmental forms of the parasitoid *S. vesparum* (larvae, pupae and adults). We also compare the chemical profiles of the parasitoid wasp adults to those of their two main host species, *Vespula vulgaris* and *Vespula germanica*. The results show differences in hydrocarbon composition of larvae, pupae and adults of *S. vesparum*. Our results also suggest a partial mimicry of each of the two host species, mostly relating to linear alkanes present in both parasitoids and the host vespid wasp species. This matching is likely due to the recycling of the prey’s hydrocarbons, as has been found in other species of parasitoids.

## 1. Introduction

Social parasites often deceive their host species using different strategies: chemical cues, mimicry, camouflage, chemical insignificance, crypsis, usurpation and weaponry [1,2]. Although multiple strategies can be employed to mimic different classes of pheromones, most of the studied interactions between arthropod associations in social insect colonies have been based on hydrocarbon mimicking [3,4]. Cuticular hydrocarbons (CHCs) have a primary function to protect against desiccation, but have acquired a communicative function in social insects. This function is the most studied mechanism used in nestmate recognition [5]. These CHCs have also recently been shown to function as queen pheromones [6]. Obligate parasites have evolved several methods to avoid olfactory CHC detection by their hosts. Strategies used by parasites can include producing low concentrations of recognition cues, demonstrating chemical insignificance, or copying chemical profiles of their host either actively or passively (chemical mimicry). In order to avoid host detection, obligate parasites often express low concentrations of recognition cues, are chemically insignificant, or copy profiles of hosts from queens or workers by chemical mimicry [1,2,7,8,9]. Whether the chemical mimicry strategy is active or passive is difficult to determine. It may be that the parasitoid is using active mimicry, where the parasite biosynthesizes the host hydrocarbon composition, or it may be that the mimicry is passive, where the parasite acquires CHC composition through contact with the host itself or nest material [2,10].

The arthropods associated with social wasps are the least studied group when compared to arthropods that live together with other social insects, such as ants or termites [4,9]. An example of the complex chemical ecology that can occur within the social insects and their visitors is the aphidiid wasp, *Lysiphlebus cardui*, that parasitizes the aphid, *Aphis fabae cirsiiacanthoidis*, and uses chemical cues to avoid aggressive behavior from the ants, *Lasius niger*, attending the aphids [11]. In honeybees, some work has been done using the ectoparasite *Varroa destructor*, showing that mites can adjust their chemical profiles depending on the host, either *Apis mellifera* or *Apis cerana*, to avoid detection [12]. Research has also shown colony-specificity in *Apis mellifera* [13]. In paper wasps, the social parasites *Polistes atrimandibularis* show lower concentrations of CHC’s, enabling them to go undetected by the host species *Polistes biglumis* [7]. The parasitic beetle *Metoecus paradoxus* uses chemical mimicry to resemble some of the hydrocarbons that occur in the host species, and are frequently found in nests of *Vespula vulgaris* [8].

Like the beetle *M. paradoxus*, *Sphecophaga vesparum vesparum* (Curtis) (Hymenoptera: Ichneumonidae) is a social parasitoid that exploits vespid wasp nests [14,15,16]. The *S. vesparum* larvae feed as an ectoparasitoid on the newly pupated forms of the wasps. There are two adult forms of *S. vesparum*: winged adults, which emerge from either thin yellow cocoons or thick yellow cocoons for overwintering, and brachypterous females, which emerge from white cocoons [14]. The species is facultative deuterotokous, where females and males can be produced without sexual fertilization of the egg [14], although males are less frequently found. In wasp species, *S. vesparum* seem to be specific to the subfamily Vespinae [17], and can be especially abundant in nests of *Vespula vulgaris* and *Vespula germanica*. This parasitoid is even used as biological control of wasp populations in invasive ranges in Australia and New Zealand [17,18,19]. To date, there has been no published characterization of cuticular compounds of *S. vesparum*, which could provide information to assist in developing alternative strategies of wasp population control.

In this study, we characterized the hydrocarbon profiles of *Sphecophaga vesparum, V. germanica* and *V. vulgaris* to determine whether the parasitoids were chemically similar to their two most common host species. We also compared chemical profiles between the parasites from different host species of wasp that were collected, and investigated whether the presence of chemical compounds could be related to the different developmental forms of the parasitoid *S. vesparum*.

## 2. Materials and Methods

### 2.1. Collection of Specimen

**Figure 1:**
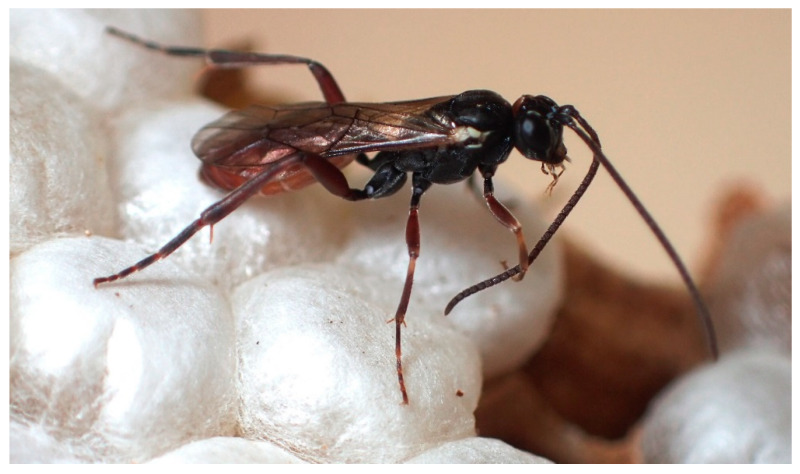
Adult female of *Sphecophaga vesparum*. Credit: Robert L. Brown.

We analyzed 47 samples in total (4 larvae of *S. vesparum* from *V. germanica* nest; 3 pupae of *S. versparum* from *V. germanica* nest; 5 big winged adults of *S. vesparum* from *V. germanica* nest; 5 small brachypterous adults of *S. vesparum* from *V. germanica* nest; 6 workers of *V. germanica*; 7 big winged adults of *S. vesparum* from *V. vulgaris* nest; 1 pupae of *S. vesparum* from *V. vulgaris* nest; 10 workers of *V. vulgaris*; 3 queens of *V. germanica* and 3 queens of *Vespula vulgaris*). The *Sphecophaga vesparum* specimens (n winged large adults = 7) from one *Vespula vulgaris* nest were collected in the United Kingdom in 2018. One pupae of *S. vesparum* (n pupae = 1) and five workers of *V. vulgaris* (n workers = 5) were collected in Belgium from another nest. *Sphecophaga vesparum* (Figure 1) have two morpho-types of females, both of which were collected from one heavily infested *Vespula germanica* nest from Belgium in 2018 (n winged large adults = 5; n brachypterous small adults = 5; n larvae = 4; n pupae = 3 and only one worker of *V. germanica,* n worker = 1). The specimens of hosts were three queens and five workers of each species of wasp, *Vespula vulgaris* (n = 3 queens from three nests and n= 5 workers from one nest) and *Vespula germanica* (n = 3 queens from three nests and n = 5 workers from one nest)*,* which were collected in the UK for the host comparisons.

### 2.2. Chemical and Statistical Analysis

Samples were extracted using 500 µL of pentane (Acros Organics, HPLC) for *Sphecophaga vesparum* and 1 ml of pentane for workers of *Vespula vulgaris* and *Vespula germanica*. After 1 min, the insects were removed from the glass vials and the extracts were evaporated under the fume hood at room temperature. The extracts were resuspended using 100 µL of hexane (HiPerSolv CHROMANORM, HPLC) for parasitoids. For wasps, we used 100 µL for workers of *V. germanica*, 150 µL for workers of *V. vulgaris* and 250 µL for both queen species. All samples were run using Gas Chromatography–Mass Spectrometry (GC-MS) (Thermo Fisher Scientific Trace 1300 connected to a Thermo Fisher Scientific ISQ mass spectrometer). The column was Restek MXT-5 (30 m, 0.25 mm and 0.25 µm film). 1 µL of each sample was injected using split-less injection at 320 °C. Initially, the temperature was held at 40 °C for 2 min, then increased to 120 °C with an increase of 20 °C/min. This was followed by an increase of 10 °C/min until 200 °C, then 7 °C/min to reach 250 °C, and a last increase of 5 °C to 350 °C/min, which was held for 4 min. The helium carrier gas had a constant flow rate of 0.9 mL/min. Alkane standards (C7 to C40 straight-chain alkanes (#49452-U, Supelco Inc., Bellefonte, PA, USA) were run as a series using the same program at three different concentrations (0.01 µg/µL, 0.005 µg/µL and 0.001 µg/µL). Peak integration was performed by integrating over total ion chromatograms using in-house developed software in R v.3.0.1. External alkane standards were used to calculate retention indices for all identified compounds based on the cubic spline method. 

Peak areas of the cuticular compounds were converted to relative amounts and a principal component analysis (PCA) was performed with the *prcomp* function of the *stats* package. The distance matrix was obtained using the *vegdist* function with Bray–Curtis dissimilarity distance. The chemical difference between parasitoids and hosts were compared using multivariate analyses (PERMANOVA) to highlight possible variations between the groups tested (origin of the individuals or species groups) with the *adonis* function in the *vegan* package in default mode with 999 permutations. We then conducted a SIMPER analysis (distance measure: Bray–Curtis, permutations equal to 999) to investigate how much each component (or peak) contributed to the observed differences in the CHC composition among groups. 

## 3. Results

**Figure 2:**
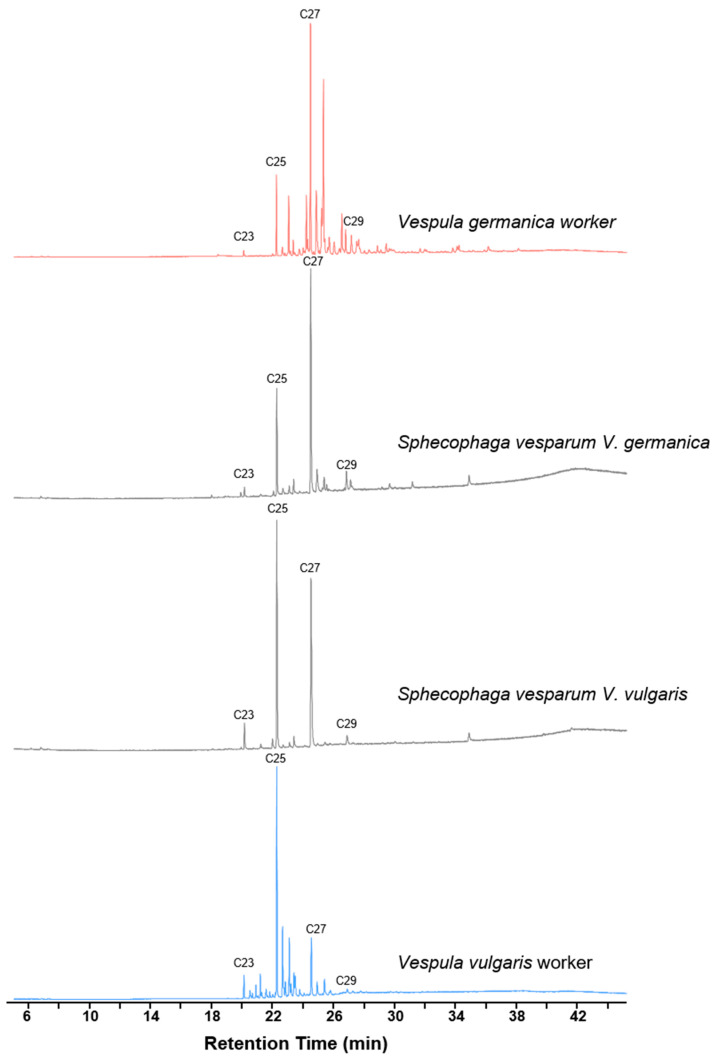
Chromatograms of the parasitoids *Sphecophaga vesparum* (in gray) and its hosts, the wasps *Vespula germanica* (in red) and *Vespula vulgaris* (in blue), indicating the identification of some linear alkanes (C23, C25, C27 and C29).

CHC profiles of different types of individuals (n = 47) were analyzed using GC-MS analysis, in which we identified 69 different compounds (Table 1 for the parasitoids and Table 2 for the hosts), mostly consisting of hydrocarbons (Table A1 in Appendix A for all identifications of compounds, retention time, retention indexes and diagnostic ions). An example of chromatograms comparing adults of *S. vesparum* parasitoids and hosts of the wasps *V. germanica* and *V. vulgaris* is shown in Figure 2.

**Figure 3:**
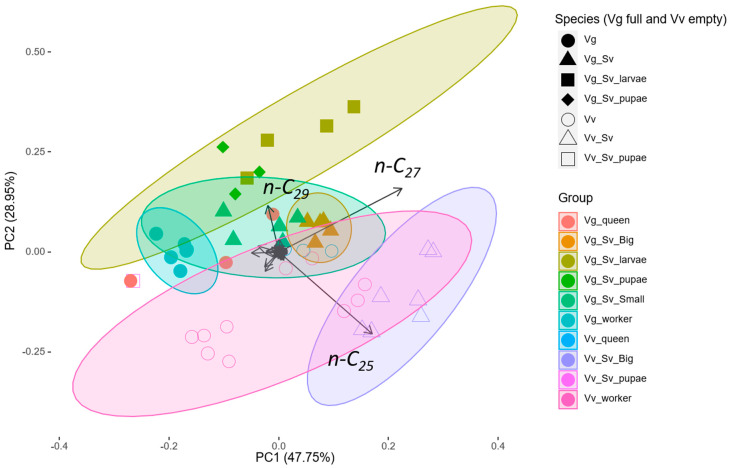
Principal component analysis (PCA) of the chemical profiles of the individuals. The host species from which the parasitoid sample was collected is indicated by a full (solid) symbol for the *Vespula germanica* nest and an empty symbol for the *Vespula vulgaris* nest. The groups represent each morpho-type by the colors. Vg: *Vespula germanica*, Vv: *Vespula vulgaris*, Sv: *Sphecophaga vesparum*, Big: winged *S. vesparum*, Small: brachypterous *S. vesparum.* Ellipses represent 95% confidence interval.

The principal component analysis of relative abundance of all compounds explained 76.70% of the total variation, in which PC1 explained 47.75% and PC2 explained 28.95% (Figure 3).

There were significant differences in the chemical profiles of all individuals collected comparing the origin of nest species, Vv or Vg (PERMANOVA, F =11.853, R^2^ = 0.208, *p* = 0.001 ***) (see also Figure 3). From SIMPER, the first five compounds responsible for the ordered cumulative contribution were *n-*C_25_ (0.197, *p* = 0.001 **), *n-*C_27_ (0.353, *p* = 0.34), 3-MeC_37_ and 11,17-diMeC_27_ (0.420, *p* = 0.22), *n-*C_29_ (0.485, *p* = 0.249) and 13-, 11-, 9-MeC_27_ (0.535, *p* = 0.002 **) (cumulative contribution for all compounds, Supplementary Table S1). When comparing between each group (Vg_Sv_larvae, Vg_Sv_pupae, Vv_Sv_pupae, Vg_Sv_Big, Vg_Sv_Small, Vv_Sv_Big, Vg_queen, Vv_queen, Vg_worker and Vv_worker), the difference was also significant (PERMANOVA, F = 10.271, R^2^ = 0.714, *p* = 0.001 ***) (cumulative contribution for all compounds, Supplementary Table S2). We then pooled together the adults, using only wasp adults (queens and workers) and adult forms of *S. vesparum* (big and small) and the difference was also significant between the adult forms (PERMANOVA, F = 13.569, R^2^ = 0.537, *p* = 0.001 ***) (cumulative contribution from the first 10 compounds are show in Table 3, all data available in Supplementary Table S3). Considering the linear alkanes, *n-*C_25_, *n-*C_27_ and *n-*C_29_, from adults of *S. vesparum* (Table 3A,B) and wasps, the SIMPER analysis showed a significant probability of getting a larger or equal average contribution in random permutation for the alkane *n-*C_25_. When comparing the hosts and its parasitoids in the SIMPER analysis (Table 3C,F), only *n-*C_27_ showed a significant probability of getting a larger or equal average contribution in random permutation in the *V. vulgaris* hosts and its parasitoids. From hosts and parasitoids collected in a different species nest (Table 3D,E), only the host *V. germanica* and parasitoids coming from *V. vulgaris* nests showed significant probability of getting a larger or equal average contribution in random permutation in the two alkanes, *n-*C_25_ and *n-*C_27_. 

## 4. Discussion

Our study shows that the commonly found parasitoid of Vespidae wasps, *Sphecophaga vesparum*, express different hydrocarbon compositions depending on whether they were found in *V. vulgaris* or *V. germanica* nests. Comparison of the relative proportions of all chemical compounds shows that there is a difference between the parasitoids and the wasps. The difference between adults of *S. vesparum* and *Vespula* wasp hosts was expressed by their different ordering of the most prevalent contribution of chemical compounds found in each. Interestingly, nest origin, *V. germanica* or *V. vulgaris* nest, separates the groups, and the alkane *n-*C_25_ showed significant probability of getting a larger or equal average contribution in random permutation. Therefore, the alkane *n-*C_25_ seem to be important in *S. vesparum* to differentiate the origin from *V*. *vulgaris* compared to those in *V*. *germanica* nests. 

The host-specific hydrocarbons do not seem to be primarily acquired through contact with the adult host, since larvae and pupae have higher amounts of the alkane *n*-C_29_ but are more likely acquired through contact with the pupal cell walls or through recycling hydrocarbons from consumed wasp pupae. We speculated that adults of the parasitoids may not be detected by the wasp host. During the pupal stage, the cocoons have a thick layer of silk that may be sufficient to protect the parasitoids during development into adults. The females can be seen on the nest walking fast and requesting trophalaxic food from the wasp larvae. Although hydrocarbon signatures of *Sphecophaga vesparum* seem to show wasp host dependency, we speculate that it is likely that chemical mimicry plays a role for *S. vesparum* to remain undetected in the colony. Partial mimicking by *S. vesparum* seems likely to be achieved via passive contact with the wasp hosts, similar to what happens for ant inquilines [20]. However, recycling of CHC by consuming the host is a mechanism that cannot be ruled out [21]. Another sphecophile, the beetle *M. paradoxus*, chemically mimics the wasp *V. vulgaris* by recycling CHC from the host [8]. The presence of *S. vesparum* has been described from nests of the vespine *Vespa orientalis* in the Middle East [16]. In this case, *Sphecophaga vesparum* would likely have (at least partially) adapted to match the chemical composition of the host due to the feeding of the wasp larvae. This is because CHC composition of the *Vespa* genus differs markedly from those of *Vespula* with a higher proportion of pentacosane and a lower proportion of heptacosane, for example [22]. Future chemical characterization of *S. vesparum* and subspecies collected from other wasp host species will provide more understanding about the chemical communication between hosts and parasitoids. 

The mite, *Varroa destructor*, which parasitizes the honeybee *Apis mellifera*, can acquire methylalkane compounds which are present on pupae of honeybees, but the mites can also lose this chemical profile once they are in isolation, indicating that mites obtain the compounds by passive mimicry [13]. As an example from ants, myrmecophiles expressed lower amounts of CHC concentrations in comparison to their host [9]. Future studies can investigate if this is also the case for sphecophiles. 

Overall, this is a first step towards understanding the chemical communication of sphecophiles of Vespidae host species. There is currently no knowledge of how parasites can locate suitable wasp nests or how they are able to infiltrate aggressive wasp colonies with usually efficient mechanisms of defense. As a next step, we suggest testing if *Sphecophaga* transplanted from one host species to another are able to change their CHC composition. Another interesting question is whether the nest invading *S. vesparum* are using chemical cues or visual cues to locate their hosts, or perhaps a combination of both. It would also be interesting to conduct bioassays to test whether the different ratio of the alkane *n-*C_25_ in *S. vesparum* is an important characteristic to stay undetected in wasp host nests. 

## 5. Conclusions

Hydrocarbon signatures of *Sphecophaga vesparum* seem to show *Vespula* wasp host dependency and it is likely that chemical mimicry plays a role in the parasite’s ability to remain undetected in the colony. Partial mimicking by *S. vesparum* seems likely to be achieved via passive contact with the wasp hosts.

## Figures and Tables

**Table 1:** List of hydrocarbons found in different life stages of the ectoparasitoid *Sphecophaga vesparum* and their relative amounts. Vg and Vv indicated if they were collected at the *Vespula germanica* or *Vespula vulgaris* nest. “Big” indicated the winged form of *S. vesparum* and “Small” indicated the brachypterous form. n = number of individuals. SD = standard deviation.

	Vg_Sv_larvae (n = 4)		Vg_Sv_pupae (n = 3)		Vg_Sv_Big (n = 5)		Vg_Sv_Small (n = 5)		Vv_Sv_Big (n = 7)		Vv_Sv_pupae (n = 1)
Identifications	Average	SD	Average	SD	Average	SD	Average	SD	Average	SD	Average
*n-*C_21_	0.35	0.10	0.51	0.34	0.51	0.10	0.75	0.12	0.47	0.33	2.60
*n-*C_22_	0.65	0.17	0.95	0.56	0.56	0.04	1.36	0.46	0.32	0.09	1.63
*n-*C_23_	0.93	0.24	1.81	0.95	1.57	0.14	2.47	0.35	4.89	2.68	1.87
11-MeC_23_	0.14	0.05	0.10	0.05	0.08	0.02	0.27	0.11	0.07	0.01	0.74
5-MeC_23_	0.11	0.02	0.07	0.03	0.09	0.02	0.29	0.10	0.08	0.01	0.99
2-MeC_23_	0.17	0.04	0.15	0.04	0.13	0.02	0.33	0.08	0.07	0.02	0.48
3-MeC_23_	0.27	0.06	0.21	0.07	0.15	0.03	0.49	0.15	0.11	0.03	1.42
5,11-diMeC_23_	0.23	0.07	0.16	0.04	0.14	0.03	0.40	0.10	0.09	0.02	1.19
*n*-C_24_	0.61	0.12	0.58	0.21	0.61	0.04	1.40	0.27	0.74	0.09	1.34
3,7-diMeC_23_	0.62	0.16	0.53	0.15	0.37	0.06	1.07	0.23	0.26	0.06	1.72
12-,11-,10-MeC_24_	0.08	0.02	0.06	0.03	0.05	0.01	0.15	0.05	0.02	0.01	0.27
4-MeC_24_	0.36	0.08	0.26	0.08	0.17	0.05	0.59	0.12	0.12	0.05	1.58
C_25:1_	0.20	0.05	0.17	0.08	0.29	0.17	0.36	0.06	0.96	1.49	1.14
4,10-; 4,14-; 4,16-diMeC_24_	0.07	0.04	0.05	0.03	0.05	0.03	0.13	0.04	0.05	0.02	0.56
*n*-C_25_	3.21	1.94	3.83	1.64	16.14	1.57	10.16	3.33	34.33	3.46	3.49
13-,11-MeC_25_	0.36	0.18	0.64	0.29	1.20	0.20	1.11	0.26	0.49	0.25	1.29
7-MeC_25_	0.15	0.08	0.23	0.09	0.37	0.04	0.40	0.11	0.15	0.08	0.61
5-MeC_25_	0.25	0.08	0.20	0.04	0.28	0.05	0.50	0.15	0.22	0.12	1.25
9,13-; 9,15-diMeC_25_	0.18	0.05	0.12	0.03	0.14	0.03	0.29	0.06	0.13	0.10	0.80
3-MeC_25_	0.39	0.25	0.86	0.36	1.67	0.23	1.26	0.28	0.80	0.38	1.31
5,9-diMeC_25_	0.26	0.15	0.27	0.08	0.28	0.05	0.48	0.16	0.32	0.19	1.56
*n-*C_26_	1.31	0.13	1.35	0.18	2.19	0.27	1.80	0.13	1.96	0.33	1.36
3,9-; 3,13-diMeC_25_	0.85	0.23	1.01	0.20	0.85	0.20	1.30	0.15	0.78	0.24	2.08
10-MeC_26_	0.48	0.15	0.65	0.11	0.74	0.15	0.84	0.17	0.28	0.11	2.11
4-MeC_26_	0.22	0.06	0.23	0.04	0.17	0.05	0.37	0.08	0.13	0.07	1.01
3-MeC_26_	0.17	0.03	0.28	0.13	0.44	0.15	0.41	0.06	0.19	0.09	1.59
4,16-; 4,18-diMeC_26_	0.07	0.03	0.06	0.04	0.08	0.08	0.11	0.04	0.02	0.01	0.48
*n-*C_27_	40.07	11.07	27.81	3.02	35.68	1.63	26.62	5.00	37.68	7.75	2.76
4,8,12-triMeC_26_	0.82	0.25	0.31	0.06	0.34	0.04	0.88	0.21	0.65	0.12	1.17
13-,11-,9-MeC_27_	1.99	1.37	6.08	1.56	8.60	1.62	3.90	0.96	0.81	0.17	2.61
5-MeC_27_	0.35	0.07	0.31	0.14	0.51	0.12	0.46	0.03	0.13	0.05	0.98
9,13-; 9,19-MeC_27_	0.60	0.30	1.21	0.22	0.76	0.10	0.51	0.09	0.17	0.05	1.27
3-MeC_27_, 11,17-diMeC_27_	1.57	1.74	4.41	0.88	4.83	0.77	2.42	0.55	0.80	0.16	2.44
*n-*C_28_	0.99	0.22	1.29	0.15	0.39	0.09	0.65	0.21	0.14	0.05	1.28
3,11-; 3,9-diMeC_27_	0.61	0.14	0.72	0.20	0.54	0.07	0.91	0.27	0.49	0.09	1.76
14-MeC_28_	0.14	0.03	0.11	0.05	0.07	0.03	0.22	0.07	0.07	0.02	0.55
10-MeC_28_	0.84	0.16	0.79	0.24	0.64	0.06	1.18	0.36	0.35	0.13	2.59
4-MeC_28_	0.54	0.17	0.55	0.05	0.49	0.11	0.99	0.25	0.35	0.12	2.06
C_29:1_	0.17	0.07	0.14	0.02	0.27	0.16	0.41	0.10	0.11	0.05	0.85
*n-*C_29_	15.79	3.60	17.71	8.92	3.25	0.99	4.57	2.82	2.07	0.63	1.71
15-, 13-, 11-MeC_29_	1.89	0.49	3.17	0.38	2.24	0.80	2.76	0.90	0.69	0.21	2.25
9,3-diMeC_29_	0.90	0.19	0.99	0.28	0.42	0.09	0.99	0.22	0.33	0.12	1.37
11,17-diMeC_29_	0.10	0.04	0.15	0.01	0.09	0.02	0.13	0.04	0.05	0.02	0.21
3-MeC_29_	1.11	0.18	1.00	0.12	0.63	0.09	1.08	0.20	0.37	0.10	1.82
*n-*C_30_	0.47	0.10	0.50	0.11	0.19	0.04	0.48	0.16	0.11	0.04	0.61
unknown3	0.56	0.13	0.47	0.03	0.39	0.07	0.87	0.14	0.27	0.07	0.97
5,9,15,19-tetraMeC_29_	0.53	0.11	0.48	0.04	0.36	0.12	0.82	0.24	0.19	0.06	1.20
C_31:1_	0.69	0.17	0.73	0.04	0.53	0.07	1.06	0.17	0.31	0.07	2.71
*n-*C_31_	2.32	0.82	2.62	1.32	0.64	0.11	1.36	0.39	0.35	0.08	2.43
15-, 13-, 11-MeC_31_	0.95	0.25	1.20	0.07	0.49	0.07	0.93	0.10	0.33	0.13	1.52
9,21-diMeC_31_	0.85	0.23	0.52	0.12	0.49	0.13	0.71	0.10	0.34	0.11	1.32
3-MeC_31,_ 5,21-diMeC_31_	2.47	1.07	0.79	0.04	0.68	0.18	1.17	0.18	0.71	0.25	1.63
*n-*C_32_	0.10	0.04	0.06	0.02	0.04	0.01	0.09	0.03	0.03	0.01	0.24
3,17-; 3,13-;3,11-diMeC_31_	0.93	0.30	0.53	0.09	0.54	0.10	1.13	0.29	0.31	0.09	2.41
14-,12-MeC_32_; 3,11,19-triMeC_32_	0.08	0.03	0.05	0.02	0.05	0.01	0.13	0.04	0.03	0.01	0.25
*n*-C_33_	0.20	0.06	0.17	0.01	0.12	0.02	0.26	0.06	0.07	0.02	0.54
15-,13-,11-MeC_33_	0.85	0.06	0.95	0.11	0.46	0.08	1.02	0.13	0.29	0.09	2.44
7,11-; 7,21-diMeC_33_	1.35	0.42	0.75	0.22	0.48	0.06	1.13	0.12	0.38	0.14	2.64
*n-*C_34_	0.10	0.02	0.12	0.02	0.08	0.02	0.16	0.03	0.05	0.01	0.41
*n-*C_35_	0.08	0.04	0.09	0.00	0.05	0.01	0.13	0.01	0.03	0.01	0.32
15-, 13-, 11-MeC_35_	0.67	0.21	0.99	0.07	0.40	0.04	0.85	0.16	0.28	0.09	2.59
2,7-diMeC_35_	0.91	0.28	0.92	0.21	0.65	0.13	1.37	0.35	0.42	0.10	4.58
unknown	3.64	1.06	4.74	1.14	3.40	0.14	6.76	1.17	1.72	0.56	2.59
*n-*C_37_	0.09	0.02	0.10	0.04	0.06	0.02	0.15	0.03	0.04	0.02	0.30
19-, 13-, 11-MeC_37_	0.29	0.10	0.40	0.04	0.27	0.05	0.49	0.18	0.12	0.06	1.43
17,21-; 13,25-; 11,21-diMeC_37_	0.43	0.01	0.48	0.03	0.36	0.06	0.67	0.14	0.19	0.06	1.57
*n-*C_38_	0.07	0.05	0.08	0.01	0.06	0.02	0.12	0.03	0.03	0.01	0.23
*n-*C_39_	0.05	0.02	0.05	0.01	0.05	0.02	0.11	0.04	0.04	0.01	0.28
*n-*C_40_	0.12	0.06	0.12	0.03	0.11	0.04	0.24	0.08	0.06	0.02	0.61

**Table 2:** List of hydrocarbons of the hosts *Vespula vulgaris* and *Vespula germanica* and their relative amounts (n = number of individuals).

	Vg_worker (n = 6)		Vv_worker (n = 10)		Vg_queen (n = 3)		Vv_queen (n = 3)	
Identifications	Average	SD	Average	SD	Average	SD	Average	SD
*n-*C_21_	0.19	0.38	0.10	0.03	0.72	1.19	0.04	0.02
*n-*C_22_	0.13	0.14	0.13	0.04	0.58	0.94	0.05	0.03
*n-*C_23_	1.74	3.63	1.89	0.65	0.79	1.02	0.48	0.07
11-MeC_23_	0.03	0.04	0.54	0.57	0.26	0.43	0.08	0.03
5-MeC_23_	0.02	0.02	0.36	0.35	0.24	0.40	0.06	0.02
2-MeC_23_	0.01	0.01	0.03	0.02	0.18	0.30	0.01	0.01
3-MeC_23_	0.06	0.02	1.02	0.93	0.44	0.62	0.21	0.06
5,11-diMeC_23_	0.03	0.02	0.26	0.24	0.35	0.56	0.08	0.04
*n*-C_24_	0.14	0.12	1.76	0.48	0.39	0.41	0.64	0.05
3,7-diMeC_23_	0.06	0.06	0.60	0.54	0.51	0.83	0.17	0.07
12-,11-,10-MeC_24_	0.01	0.01	0.01	0.00	0.10	0.17	0.01	0.00
4-MeC_24_	0.08	0.01	0.57	0.45	0.66	0.95	0.12	0.02
C_25:1_	0.34	0.14	0.33	0.27	0.57	0.52	0.12	0.03
4,10-; 4,14-; 4,16-diMeC_24_	0.03	0.01	0.37	0.34	0.22	0.33	0.08	0.02
*n*-C_25_	6.62	1.07	23.09	5.69	7.99	4.06	18.47	2.14
13-,11-MeC_25_	1.44	0.59	6.07	5.36	0.79	0.49	1.42	0.37
7-MeC_25_	0.26	0.12	0.76	0.59	0.32	0.32	0.22	0.02
5-MeC_25_	0.30	0.04	1.68	0.93	0.56	0.61	0.41	0.11
9,13-; 9,15-diMeC_25_	0.22	0.09	0.16	0.12	0.31	0.32	0.09	0.02
3-MeC_25_	5.40	2.06	6.04	1.69	6.22	4.98	3.45	0.36
5,9-diMeC_25_	0.50	0.15	1.38	1.11	0.69	0.53	0.49	0.05
*n-*C_26_	1.14	0.39	3.64	0.97	1.99	0.80	4.84	0.50
3,9-; 3,13-diMeC_25_	0.41	0.12	2.41	1.90	0.92	0.91	0.96	0.12
10-MeC_26_	0.99	0.26	1.06	0.86	0.93	0.85	0.40	0.11
4-MeC_26_	0.07	0.02	0.16	0.08	0.34	0.51	0.05	0.01
3-MeC_26_	6.18	3.09	0.62	0.41	3.03	1.58	0.60	0.29
4,16-; 4,18-diMeC_26_	0.27	0.11	0.17	0.13	0.23	0.14	0.07	0.02
*n-*C_27_	15.29	4.11	20.00	11.69	19.13	15.69	30.89	3.28
4,8,12-triMeC_26_	0.29	0.09	0.10	0.03	0.55	0.61	0.12	0.05
13-,11-,9-MeC_27_	9.34	0.59	2.81	1.96	3.67	0.95	1.53	0.49
5-MeC_27_	0.32	0.06	0.32	0.15	0.48	0.53	0.13	0.04
9,13-; 9,19-MeC_27_	5.51	2.17	0.35	0.18	2.28	0.85	0.19	0.06
3-MeC_27_, 11,17-diMeC_27_	15.46	6.00	5.47	2.57	12.35	8.37	7.68	0.81
*n-*C_28_	0.87	0.08	0.94	0.65	1.16	0.22	1.76	0.27
3,11-; 3,9-diMeC_27_	1.86	0.64	1.59	0.57	1.36	0.14	0.81	0.10
14-MeC_28_	0.02	0.02	0.04	0.02	0.15	0.17	0.02	0.02
10-MeC_28_	1.62	0.44	0.39	0.21	1.38	1.11	0.35	0.06
4-MeC_28_	0.86	0.27	0.56	0.10	1.01	0.60	0.39	0.14
C_29:1_	0.53	0.24	0.35	0.18	0.56	0.33	0.27	0.16
*n-*C_29_	5.64	9.47	3.07	1.51	2.48	0.37	6.08	1.28
15-, 13-, 11-MeC_29_	2.37	1.01	1.09	0.67	1.61	0.88	0.85	0.21
9,3-diMeC_29_	1.41	0.36	0.48	0.27	1.62	0.21	0.49	0.09
11,17-diMeC_29_	0.36	0.13	0.07	0.06	0.35	0.14	0.14	0.02
3-MeC_29_	1.51	0.62	1.27	0.39	2.05	0.12	4.55	0.89
*n-*C_30_	0.13	0.06	0.18	0.14	0.57	0.31	0.24	0.05
unknown3	0.32	0.05	0.57	0.20	0.85	0.61	0.28	0.04
5,9,15,19-tetraMeC_29_	0.45	0.13	0.30	0.19	0.94	0.85	0.26	0.13
C_31:1_	0.69	0.36	0.57	0.23	1.19	1.34	0.73	0.37
*n-*C_31_	0.81	1.11	0.49	0.20	1.39	1.63	0.60	0.06
15-, 13-, 11-MeC_31_	1.16	0.59	0.36	0.18	1.16	0.93	0.58	0.16
9,21-diMeC_31_	0.72	0.22	0.34	0.28	0.87	0.57	1.09	0.18
3-MeC_31,_ 5,21-diMeC_31_	0.55	0.19	0.51	0.22	1.00	0.82	2.07	0.49
*n-*C_32_	0.03	0.01	0.03	0.02	0.08	0.09	0.04	0.01
3,17-; 3,13-;3,11-diMeC_31_	0.32	0.17	0.39	0.16	0.83	0.98	0.30	0.07
14-,12-MeC_32_; 3,11,19-triMeC_32_	0.03	0.02	0.03	0.02	0.12	0.17	0.01	0.01
*n*-C_33_	0.08	0.13	0.06	0.02	0.20	0.29	0.04	0.00
15-,13-,11-MeC_33_	0.69	0.61	0.27	0.10	1.05	1.24	0.31	0.04
7,11-; 7,21-diMeC_33_	0.80	0.42	0.38	0.25	1.31	1.44	1.74	0.42
*n-*C_34_	0.04	0.05	0.04	0.02	0.15	0.19	0.06	0.01
*n-*C_35_	0.02	0.02	0.02	0.01	0.14	0.21	0.02	0.01
15-, 13-, 11-MeC_35_	0.60	0.30	0.27	0.08	0.85	0.89	0.21	0.07
2,7-diMeC_35_	1.37	0.35	0.39	0.19	1.81	1.67	1.15	0.26
unknown	0.39	0.46	0.30	0.09	1.01	1.35	0.22	0.10
*n-*C_37_	0.02	0.02	0.03	0.01	0.09	0.12	0.02	0.01
19-, 13-, 11-MeC_37_	0.15	0.05	0.10	0.05	0.43	0.64	0.05	0.04
17,21-; 13,25-; 11,21-diMeC_37_	0.62	0.17	0.18	0.07	0.97	1.05	0.10	0.06
*n-*C_38_	0.03	0.03	0.03	0.02	0.15	0.21	0.02	0.01
*n-*C_39_	0.02	0.02	0.03	0.02	0.13	0.20	0.01	0.01
*n-*C_40_	0.04	0.04	0.05	0.04	0.20	0.29	0.02	0.02

**Table 3:** Contribution of compounds discriminating the adult groups using SIMPER Bray–Curtis dissimilarities (999 permutations). (A) Vg_Sv versus Vv_Sv, (B) Vg versus Vv, (C) Vg versus Vg_Sv, (D) Vg versus Vv_Sv, (E) Vv versus Vg_Sv and (F) Vv versus Vv_Sv (Vg: *Vespula germanica,* Sv: *Sphecophaga vesparum*, Vv: *Vespula vulgaris*). The percentage of contribution for each chemical compound that explains the similarity between the compared groups is indicated. The compounds were classified from the highest to the lowest percentage of contribution, shown in the cumulative contribution (%). The *p*-values from SIMPER were obtained when permutations were calculated (Permutation *p*-value as the probability of getting a larger or equal average contribution in random permutation of the group factor).

	**(A)**	**Vg_Sv versus Vv_Sv**				**(B)**	**Vg versus Vv**		
	**Compound**	**Cumulative**	***p*-Value**			**Compound**	**Cumulative**	***p*-Value**	
1	*n-*C_25_	0.31	0.001	***	1	*n-*C_25_	0.15	0.007	**
2	*n-*C_27_	0.45	0.991		2	*n-*C_27_	0.29	0.584	
3	13-, 11-, 9-MeC_27_	0.53	0.004	**	3	3-MeC27, 11-17-diMeC_27_	0.39	0.001	***
4	unknown	0.58	0.008	**	4	13-, 11-, 9-MeC_27_	0.45	0.003	**
5	*n-*C_23_	0.62	0.056		5	3-meC_26_	0.50	0.001	***
6	3-MeC27, 11-17-diMeC_27_	0.67	1.000		6	13-, 11-MeC_25_	0.54	0.021	*
7	*n-*C_29_	0.69	0.692		7	*n-*C_29_	0.59	0.088	
8	15-, 13-, 11-MeC_29_	0.72	0.001	***	8	9,13-; 9,19-diMeC27	0.63	0.001	***
9	C_25:1_	0.73	0.054		9	3-MeC_25_	0.66	0.892	
10	3-MeC_25_	0.74	0.974		10	*n*-C_26_	0.69	0.001	***
	**(C)**	**Vg versus Vg_Sv**				**(D)**	**Vg versus Vv_Sv**		
	**Compound**	**Cumulative**	***p*-Value**			**Compound**	**Cumulative**	***p*-Value**	
1	*n-*C_27_	0.18	0.068		1	*n-*C_25_	0.23	0.001	***
2	3-MeC27, 11-17-diMeC_27_	0.31	0.001	***	2	*n-*C_27_	0.41	0.001	***
3	*n-*C_25_	0.39	1.000		3	3-MeC27, 11-17-diMeC_27_	0.53	0.001	***
4	3-meC_26_	0.45	0.001	***	4	13-, 11-, 9-MeC_27_	0.59	0.001	***
5	unknown	0.50	0.001	***	5	3-meC_26_	0.63	0.001	***
6	3-MeC_25_	0.55	0.003	**	6	3-MeC_26_	0.67	0.002	**
7	*n-*C_29_	0.60	0.210		7	*n*-C_23_	0.71	0.001	***
8	9,13-; 9,19-diMeC27	0.64	0.001	***	8	9,13-; 9,19-diMeC_27_	0.74	0.001	***
9	13-, 11-, 9-MeC_27_	0.68	0.929		9	*n*-C_29_	0.77	0.445	
10	*n-*C_23_	0.71	0.486		10	15-, 13-, 11-MeC_29_	0.78	0.058	
	**(E)**	**Vv versus Vg_Sv**				**(F)**	**Vv versus Vv_Sv**		
	**Compound**	**Cumulative**	***p*-Value**			**Compound**	**Cumulative**	***p*-Value**	
1	*n-*C_27_	0.16	0.844		1	*n-*C_27_	0.21	0.041	*
2	*n-*C_25_	0.29	0.995		2	*n-*C_25_	0.38	0.312	
3	unknown	0.36	0.001	***	3	3-MeC27, 11-17-diMeC_27_	0.45	0.860	
4	13-, 11-meC_25_	0.42	0.009	**	4	3-MeC_25_	0.52	0.001	***
5	13-, 11-, 9-meC_27_	0.48	0.206		5	13-, 11-meC_25_	0.58	0.038	*
6	3-MeC_25_	0.53	0.005	**	6	*n*-C_23_	0.63	0.008	**
7	3-MeC27, 11-17-diMeC_27_	0.58	1.000		7	*n*-C_29_	0.65	0.639	
8	*n*-C_29_	0.61	0.657		8	*n*-C_26_	0.68	0.004	**
9	*n*-C_26_	0.63	0.001	***	9	15-, 13-, 11-MeC_29_	0.70	1.000	
10	15-, 13-, 11-MeC_29_	0.65	0.003	**	10	3-MeC_29_	0.73	0.015	*

*, *p* < 0.05; **, *p* < 0.01; ***, *p* < 0.005.

## Supplementary Materials

The following are available online at https://www.mdpi.com/2075-4450/11/5/268/s1, Table S1: Contribution of compounds discriminating the origin of the samples, if they were collected from *Vespula germanica* (Vg) or *Vespula vulgaris* (Vv) using SIMPER Bray–Curtis dissimilarities (999 permutations). The percentage of contribution of each chemical compound that explains the similarity between the two groups is indicated. The compounds were classified from the highest to the lowest percentage of contribution, shown in the cumulative contribution (%). The *p*-values from SIMPER were obtained when permutations were calculated (Permutation *p*-value as the probability of getting a larger or equal average contribution in random permutation of the group factor). * *p* < 0.05, ** *p* < 0.01, *** *p* < 0.001. Table S2: Contribution of compounds discriminating all group of the samples Vg_Sv_larvae, Vg_Sv_pupae, Vv_Sv_pupae, Vg_Sv_Big, Vg_Sv_Small, Vv_Sv_Big, Vg_queen, Vv_queen, Vg_worker and Vv_worker using SIMPER Bray–Curtis dissimilarities (999 permutations) (Vg: *Vespula germanica*, Sv: *Sphecophaga vesparum*, Vv: *Vespula vulgaris*, Big: winged *S. vesparum*, Small: brachypterous *S. vesparum*). Table S3: Contribution of compounds discriminating the adult groups Vg, Vv, Vg_Sv, Vv_Sv using SIMPER Bray–Curtis dissimilarities (999 permutations).

## Appendix A

**Table A1:** Retention time (RT), Retention index (RI) and identification of compounds for all samples.

	RT (min)	RI	Identifications	Diagnostic Ions
1	17.92	2100	*n-*C_21_	296
2	19.01	2200	*n-*C_22_	310
3	20.09	2298	*n-*C_23_	324
4	20.48	2334	11-MeC_23_	168, 169, 196, 197
5	20.64	2349	5-MeC_23_	84, 85, 280, 281
6	20.71	2356	2-MeC_23_	42, 43, 322, 323
7	20.88	2371	3-MeC_23_	56, 57, 308, 309
8	20.99	2382	5,11-diMeC_23_	84 196, 183, 295
9	21.16	2398	*n*-C_24_	338
10	21.25	2406	3,7-diMeC_23_	56, 127, 252, 323
11	21.40	2420	12-,11-,10-MeC_24_	197, 183/168, 210/154, 224,225
12	21.79	2456	4-MeC_24_	70, 71, 308, 309
13	21.98	2473	C_25:1_	83, 97, 111
14	22.16	2490	4,10-; 4,14-; 4,16-diMeC_24_	70, 169, 224, 323/ 168, 225/ 140, 253, 337
15	22.25	2499	*n*-C_25_	352
16	22.63	2533	13-,11-MeC_25_	168, 196, 224
17	22.70	2540	7-MeC_25_	112, 281
18	22.81	2550	5-MeC_25_	85, 309
19	22.97	2564	9,13-; 9,15-diMeC_25_	140, 168, 211, 267/239, 267
20	23.06	2573	3-MeC_25_	57, 337
21	23.16	2582	5,9-diMeC_25_	85, 155, 252, 323
22	23.36	2600	*n-*C_26_	366
23	23.44	2607	3,9-; 3,13-diMeC_25_	155, 252, 351/196, 211
24	23.74	2633	10-MeC_26_	154, 253
25	23.86	2644	4-MeC_26_	71, 337
26	24.22	2676	3-MeC_26_	56, 301, 351
27	24.37	2689	4,16-; 4,18-diMeC_26_	70, 168, 253, 351/140, 281
28	24.50	2700	*n-*C_27_	380
29	24.68	2716	4,8,12-triMeC_26_	141, 224, 211, 295, 365
30	24.87	2733	13-,11-,9-MeC_27_	197, 224/168, 252/141, 281
31	25.07	2750	5-MeC_27_	85, 337
32	25.22	2763	9,13-; 9,19-MeC_27_	140, 211, 295/140, 295
33	25.35	2774	3-MeC_27_, 11,17-diMeC_27_	57, 366/ 168, 267
34	25.65	2801	*n-*C_28_	394
35	25.72	2807	3,11-; 3,9-diMeC_27_	155, 183 252, 379/155, 281, 380
36	25.88	2821	14-MeC_28_	210, 211, 224
37	26.04	2834	10-MeC_28_	155, 280
38	26.38	2864	4-MeC_28_	70, 364
39	26.65	2887	C_29:1_	83, 97, 111
40	26.81	2902	*n-*C_29_	408
41	27.17	2933	15-, 13-, 11-MeC_29_	224, 225/196, 252/168, 281
42	27.53	2965	9,3-diMeC_29_	140, 211
43	27.60	2970	11,17-diMeC_29_	168, 295
44	27.68	2978	3-MeC_29_	56, 393
45	27.97	3003	*n-*C_30_	422
46	28.03	3008	unknown3	
47	28.34	3035	5,9,15,19-tetraMeC_29_	84, 155, 182, 239, 253, 337, 407
48	28.89	3083	C_31:1_	83, 97, 111
49	29.12	3104	*n-*C_31_	436
50	29.47	3135	15-, 13-, 11-MeC_31_	224, 252/196, 280/ 168, 308
51	29.83	3166	9,21-diMeC_31_	140, 168, 323, 351
52	29.99	3181	3-MeC_31,_ 5,21-diMeC_31_	56, 420/84, 168, 323
53	30.19	3198	*n-*C_32_	450
54	30.21	3200	3,17-; 3,13-;3,11-diMeC_31_	224, 267, 435/211, 280/ 183, 308
55	30.51	3227	14-,12-MeC_32_; 3,11,19-triMeC_32_	183, 196, 309, 323, 450
56	31.40	3306	*n*-C_33_	464
57	31.72	3336	15-,13-,11-MeC_33_	224, 280/ 197, 309/ 168, 169, 337
58	32.07	3367	7,11-; 7,21-diMeC_33_	112, 183, 336/ 112, 196, 323
59	32.43	3400	*n-*C_34_	478
60	33.52	3501	*n-*C_35_	492
61	33.85	3532	15-, 13-, 11-MeC_35_	224, 308/ 196, 337/168, 465
62	34.24	3570	2,7-diMeC_35_	113, 434
63	34.90	3632	unknown	
64	35.58	3699	*n-*C_37_	520
65	35.93	3733	19-, 13-, 11-MeC_37_	280, 281/ 196, 365/ 168, 393
66	36.20	3759	17,21-; 13,25-; 11,21-diMeC_37_	252, 323/196, 379/ 168, 252, 323
67	36.62	3801	*n-*C_38_	534
68	37.62	3901	*n-*C_39_	548
69	38.64	4003	*n-*C_40_	562
